# Detection of Subclinical Atherosclerosis in Asymptomatic Subjects Using Ultrasound Radiofrequency-Tracking Technology

**DOI:** 10.1371/journal.pone.0111926

**Published:** 2014-11-04

**Authors:** Lili Niu, Yanling Zhang, Long Meng, Yang Xiao, Kelvin K. L. Wong, Derek Abbott, Hairong Zheng, Rongqin Zheng, Ming Qian

**Affiliations:** 1 Paul C. Lauterbur Research Center for Biomedical Imaging, Institute of Biomedical and Health Engineering, Shenzhen Institutes of Advanced Technology, Chinese Academy of Sciences, Shenzhen, China; 2 Department of Ultrasound, Third Affiliated Hospital of Sun Yat-sen University, Guangzhou, China; 3 Centre for Biomedical Engineering, and School of Electrical & Electronic Engineering, University of Adelaide, Adelaide, Australia; University of Bologna, Italy

## Abstract

**Objective:**

Atherosclerosis is a chronic and systemic disease and its developmental process involves the synergism of multiple risk factors such as hypertension, dyslipidemia, diabetes, obesity and smoking. The diagnosis of subclinical atherosclerosis is essential for strategic guidance towards suitable treatments and efficient prevention against acute cardiovascular events. This study employed ultrasound radiofrequency (RF) tracking technology to characterize human carotid arteries *in vivo* in terms of intima-media thickness (IMT) and artery stiffness, and evaluated the statistical correlation between carotid IMT and stiffness, and the number of risk factors for atherosclerosis.

**Methods:**

A total of 160 asymptomatic subjects were enrolled. Ultrasound RF-tracking technology was employed to acquire carotid IMT and stiffness parameters: maximum IMT (^MAX^IMT), RF Quality IMT (^RF^QIMT), distensibility coefficient (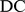), compliance coefficient (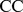), 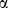
index, 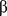 index and local pulse wave velocity (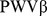). The subjects were categorized in four groups in terms of the number of risk factors: ‘zero’, ‘single’, ‘double’, and ‘multiple’, and statistical analyses of carotid IMT and stiffness parameters were performed between these different groups.

**Results:**

The subjects (*n* = 145) with ^MAX^IMT smaller than 1.0 mm matched the IMT criteria for non-atherosclerosis and were named as NA-subjects. Spearman’s rho correlation analysis of the whole group and the NA-subjects both showed that ^MAX^IMT correlated positively with ^RF^QIMT, 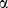, 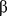, and 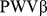, and negatively with 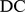 and 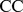 (*p*<0.01). The analysis of covariance of NA-subjects showed significant differences between subjects with and without risk factors, and also showed significant differences between the ‘zero’, ‘single’, ‘double’, and ‘multiple’ groups.

**Conclusions:**

The carotid IMT and stiffness parameters obtained by the ultrasound RF-tracking technology were demonstrated to possess significant statistical correlation with the number of risk factors from 160 subjects, and these anatomical and mechanical parameters may potentially be used together with the IMT criteria to support subclinical atherosclerosis diagnosis.

## Introduction

Atherosclerosis is a leading cause of cardiovascular death due to the increasing prevalence of the disease and the impact of risk factors such as hypertension, dyslipidemia, diabetes, obesity and smoking [1]–[3]. Subclinical atherosclerotic changes on arterial walls involve intima-media thickening and decreased arterial elasticity. Carotid intima-media thickness (IMT) and arterial stiffness, pulse wave velocity, arterial compliance and distensibility, and the elastic modulus of the vessel wall, are good indicators of early atherosclerosis and potential cardiovascular events [4]–[7]. A quantitative method for cumulative risk assessment of asymptomatic subjects needs to be developed by measuring these indicators and exploring the statistical relationship between these indicators and the atherosclerosis risk factors, so as to make it possible to achieve early detection of subclinical atherosclerosis in asymptomatic subjects and perform preventive and effective treatment.

The carotid IMT has been mostly measured online or offline from ultrasound *B*-mode images by manual, semi-manual or automated detection of the lumen-intima and media-adventitia interfaces over an artery segment [5], [8]. Arterial stiffness can be estimated by measuring the diameter change during the cardiac cycle from *B*-mode images in conjunction with the local pulse pressure [9]. However, these methods are highly machine- and operator- dependent, and are time-consuming. Ultrasound radiofrequency (RF)-tracking technology provides automated measurements of carotid IMT and stiffness, and is unaffected by the *B*-mode image quality and less dependent on the operator. This technology can precisely detect the vessel wall along each RF-vector line in real time, and tracks the diameter waveform as a function of time using a complex cross-correlation model [10], and acquires the cardiac cycle based on the waveform periodicity. When the blood pressure data are employed, this technology can automatically calculate relevant indicators that reflect change in arterial elasticity. Ultrasound RF-tracking technology has been successfully applied to assess cardiovascular risk in isolated systolic hypertension patients, in hypertriglyceridemic subjects [11], in rheumatology patients [12], in obese children [13], and in women with preeclampsia [14]. But to the best of our knowledge, no study has investigated the relationship between carotid IMT and stiffness and the number of risk factors for atherosclerosis using this technology.

Therefore, the aim of the present investigation was to assess the feasibility of using ultrasound RF-tracking technology to detect subclinical atherosclerosis by studying the correlation between carotid IMT and stiffness and the number of risk factors for atherosclerosis.

## Subjects and Methods

### A. Subjects

The study population was composed of 160 subjects, of which 112 subjects had one or more risk factors for atherosclerosis (54 men and 58 women; age 17 to 79 years) and 48 subjects had no risk factor (17 men and 31 women; age 21 to 66 years). Information regarding age, gender, blood pressure, body mass index (BMI), history of smoking, total cholesterol (TC), low density lipoprotein cholesterol (LDL-C), high density lipoprotein cholesterol (HDL-C), triglyceride (TG), fasting plasma glucose (FPG), and medication use was collected for all subjects. The study protocol was approved by the Institutional Review Board of the Third Affiliated Hospital of Sun Yat-sen University, and written informed consent was obtained from subjects or on the behalf of minors/children subjects from their guardians.

### B. Definition of risk factors

The criteria used were guided by Adult Treatment Panel III and the World Health Organization [15], [16]. Hypertension was defined as resting systolic blood pressure (SBP) ≥140 mmHg and/or diastolic blood pressure (DBP) ≥90 mmHg and/or taking antihypertensive drugs. Pulse pressure (PP) is the difference between the SBP and DBP. Dyslipidemia was defined as taking antihyperlipidemic drugs or having one or more of the following: TC≥5.2 mmol/L, LDL-C≥3.4 mmol/L, HDL-C≤1.0 mmol/L, or TG≥1.70 mmol/L. Diabetes was defined as FPG≥7.0 mmol/L or the use of anti-diabetic medication. An obesity condition was defined as a BMI≥**30.0 **kg/m^2^. Smoking status was ascertained by a questionnaire to classify each subject as a non-smoker, former smoker, or current smoker. For the purpose of the present study, “ever-smoker” status (former or current) was used. Subjects were classified as having zero (*n* = 48), single (*n* = 22), double (*n* = 52), and multiple (*n* = 38) risk factors. The subjects’ characteristics are shown in Table 1.

**Table 1:** Characteristics of subjects with different numbers of risk factors.

Characteristics	Zero (*n* = 48)	Single (*n* = 22)	Double (*n* = 52)	Multiple (*n* = 38)
Age (years)	37.37±14.21	46.09±16.69	53.75±10.26	57.21±12.45
Male gender (%)	35%,17/48	41%,9/22	42%,22/52	61%,23/38
SBP (mmHg)	115.00±9.90	121.64±11.40	125.13±15.54	135.39±18.50
DBP (mmHg)	73.13±6.31	75.91±8.14	76.00±8.23	81.29±12.75
PP (mmHg)	41.88±8.97	45.73±9.12	49.13±12.28	54.11±13.92
Height (cm)	163.56±7.33	164.05±7.03	162.54±8.44	163.84±8.51
Weight (kg)	56.58±9.56	61.14±11.34	64.02±10.05	70.11±12.37
BMI (kg/m^2^)	21.04±2.44	22.63±3.26	24.12±2.36	26.05±3.69
TC (mmol/L)	4.24±0.76	5.24±1.83	5.02±1.01	4.84±0.93
TG (mmol/L)	1.02±0.35	2.36±3.07	1.83±1.40	2.91±3.04
HDL-C (mmol/L)	1.37±0. 33	1.26±0.32	1.12±0.23	1.06±0.28
LDL-C (mmol/L)	2.46±0.57	3.14±1.17	3.31±0.89	3.04±0.74
FBG (mmol/L)	5.00±0.54	7.23±5.72	9.18±6.17	9.88±4.46
Hypertension (%)	0,0/48	13.6%,3/22	38.5%,20/52	76.3%,29/38
Dyslipidemia (%)	0,0/48	54.5%, 12/22	76.9%,40/52	97.4%,37/38
Diabetes (%)	0,0/48	22.7%,5/22	69.2%,36/52	86.8%,33/38
Obesity (%)	0,0/48	0, 0/22	0, 0/52	15.8%,6/38
Smoking (%)	0,0/48	9%,2/22	17.3%,9/52	50.0%,19/38
^MAX^IMT (mm)	0.460±0.15	0.556±0.16	0.723±0.22	0.816±0.30

SBP, systolic blood pressure; DBP, diastolic blood pressure; PP, pulse pressure; BMI, body mass index; TC, total cholesterol; TG, triglycerides; HDL-C, high-density lipoprotein cholesterol; LDL-C, low-density lipoprotein cholesterol; FBG, fasting blood glucose; ^MAX^IMT, maximum intima-media thickness.

### C. Carotid ultrasonography

The subjects underwent ultrasound examination on an Esaote MyLab90 platform (Esaote Medical Systems, Italy) using a high-resolution 4–13 MHz linear-array transducer (LA523) by an experienced angiographer. The system employed dedicated software RF-tracking technology to obtain RF Quality IMT (RF-QIMT) and RF Quality Arterial Stiffness (RF-QAS). Detailed description of the measurement process for RF-QIMT and RF-QAS technology can be found in [17].

**Figure 1:**
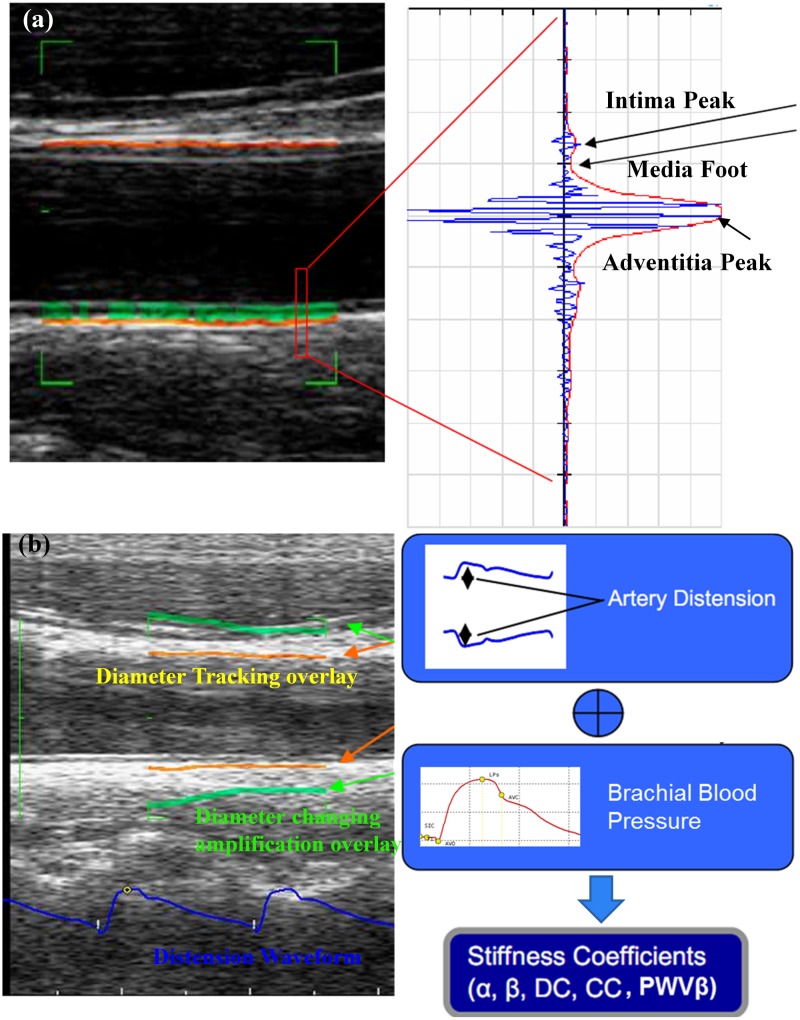
The illustration for the ultrasound radiofrequency (RF)-tracking technology. (a) The diagram for the RF-QIMT technology. A green box is chosen to define the region of interest (ROI). On the right it shows a typical RF signal that corresponds to the red rectangle in the ROI, with the RF raw signal denoted in blue, and the RF envelop denoted in red. The ‘Intima Peak’, ‘Media Foot’, and ‘Adventitia Peak’ are identified in the RF envelop in order to determine the interfaces between the intima, the media, and the adventitia in the artery. The green line inside the ROI denotes the intima and the orange line denotes the adventitia. The intima-media thickness (IMT) can therefore be obtained. (b) The diagram for the RF-QAS technology. In the same ROI, the diameter waveform is tracked as a function of time using a complex cross-correlation model, and the cardiac cycle is acquired based on the waveform periodicity. Combining with the brachial blood pressure information (systolic blood pressure and diastolic blood pressure), five arterial stiffness coefficients can be calculated, including distensibility coefficient (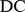), compliance coefficient (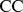), 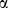 index, 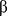 index and local pulse wave velocity (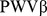).

All measurements were taken in the supine position with head elevation of ≤45° and a side tilt of 30° to the right. Note that *B*-mode examinations were undertaken on the distal wall of the common carotid artery (CCA) on the optimal image and IMT were obtained. The CCA, carotid bulb and portions of the internal and external carotid arteries on the left side were scanned. The scan encompassed the region between 30 mm proximal to the beginning of the dilation of the bifurcation bulb and 15 mm distal to the CCA flow divider. We defined the maximum IMT (^MAX^IMT) as the thickest IMT in the scanned regions. The distal 10 mm of the CCA just proximal to the bulb was measured by the ultrasound RF-tracking technology. Here, ^RF^QIMT was defined as IMT measured by RF-QIMT technology, as shown in Fig. 1 (a).

The RF-QAS technology provides a list of stiffness parameters that are calculated by combining the measured distension waveform with the brachial blood pressure that can be obtained externally via an automated system or a standard cuff, as shown in Fig. 1 (b). We define the parameters obtained by RF-QAS technology as ^RF^QAS, which can be expressed as distensibility coefficient (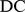), compliance coefficient (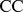), 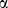 index, 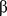 index and local pulse wave velocity (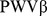).

These parameters were calculated using the following formulas [18], [19], in which 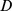 and 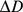 are the diastolic diameter and the change of diameter in systole, respectively, 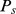 and 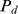 are SBP and DBP, 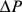 is PP, and 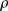 is the blood density.

The parameter 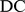 is the absolute change in vessel diameter during systole for a giving pressure change and can be calculated as following,
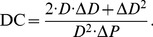
(1)


The parameter 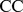 is the relative change in vessel diameter during systole for a given pressure change and is calculated as follows,
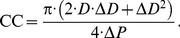
(2)


The parameter 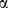 relates cross sectional area change to driving pressure, and is calculated as follows,
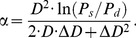
(3)


The parameter 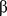 represents the degree of arteriosclerosis, and it increases in the presence of arteriosclerosis,
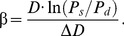
(4)


The parameter 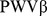 is the travel speed of pulse wave. The stiffer the artery is, the higher the 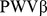 will be,
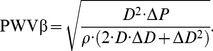
(5)


### D. Statistical analyses

Spearman’s rho correlation analysis was used to examine the bivariate relationships between ^MAX^IMT and the parameters measured by ultrasound RF-tracking technology. The analysis of covariance (ANCOVA), adjusted with age as a covariate, was used to examine which parameters were independent predictors of patients at high risk of atherosclerosis. Stepwise multiple linear regression analysis was performed to evaluate the independent factors that were significantly related to arterial elasticity. A *p* value less than 0.05 was accepted as indicating statistical significance. All statistical analyses were performed using the Statistical Package for Social Sciences statistical software package, version 17.0 (SPSS Inc.).

## Results

The Spearman’s rho correlation analysis enables the comparison between ^MAX^IMT and the parameters measured by the ultrasound RF-tracking technology, and the results are listed in Table 2. When all the subjects (*n* = 160) were analyzed, ^MAX^IMT correlated positively with ^RF^QIMT (Spearman’s rho = 0.792, *p*<0.001), 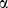 (Spearman’s rho = 0.531, *p*<0.001), 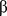 (Spearman’s rho = 0.537, *p*<0.001) and 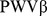 (Spearman’s rho = 0.538, *p*<0.001) and negatively with 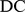 (Spearman’s rho =  −0.503, *p*<0.001) and 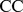 (Spearman’s rho =  −0.338, *p*<0.001). Among all subjects, 145 subjects had a ^MAX^IMT smaller than 1.0 mm thus matched the IMT criteria for non-atherosclerosis and were named as NA-subjects. When NA-subjects were analyzed, significant correlations can also be found between ^MAX^IMT and ^RF^QIMT (Spearman’s rho = 0.749, *p*<0.001), 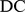 (Spearman’s rho =  −0.539, *p*<0.001), 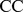 (Spearman’s rho =  −0.409, *p*<0.001), 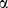 (Spearman’s rho = 0.574, *p*<0.001), 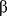 (Spearman’s rho = 0.580, *p*<0.001) and 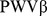 (Spearman’s rho = 0.588, *p*<0.001).

**Table 2:** The Spearman’s rho Correlation Analysis between ^MAX^IMT and ^RF^QIMT, DC, CC, α, β, PWVβ measured by the ultrasound RF-tracking technology.

		^RF^QIMT (mm)	DC (1/kPa)	CC (mm^2^/kPa)	α index	β index	PWVβ (m/s)
All subjects (*n* = 160)							
^MAX^IMT	Spearman’s rho	0.792**	–0.503**	–0.338**	0.531**	0.537**	0.538**
	*p* value	<0.001	<0.001	<0.001	<0.001	<0.001	<0.001
Subjects with ^MAX^IMT<1.0 mm (*n* = 145)							
^MAX^IMT	Spearman’s rho	0.749**	–0.539**	–0.409**	0.574**	0.580**	0.588**
	*p* value	<0.001	<0.001	<0.001	<0.001	<0.001	<0.001

DC, distensibility coefficient; CC, compliance coefficient. **p<0.01.

The NA-subjects were also analyzed by ANCOVA, adjusted with age as a covariate, and ^RF^QIMT, 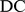, 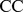, 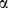, 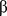 and 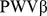 were used as input values as possible predictors. Table 3 shows significant differences in all parameters (*p*<0.05) between subjects without risk factor (*n* = 47) and subjects with one or more risk factors (*n* = 98). Comparatively, ^RF^QIMT, 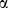, 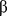 and 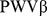 were significantly higher, and 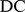 and 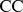 were significantly lower in subjects with risk factors than in subjects without risk factor.

**Table 3:** The ANCOVA of ^RF^QIMT, DC, CC, α, β, PWVβ in two groups of non-atherosclerosis subjects (^MAX^IMT<1.0 mm).

Parameters	Subjects without riskfactor (*n* = 47)	Subjects with risk factors (*n* = 98)	F value	*p* value
^RF^QIMT (mm)	0.560±0.019	0.608±0.012	4.079	0.045
DC (1/kPa)	0.024±0.002	0.017±0.001	7.524	0.007
CC (mm^2^/kPa)	0.900±0.055	0.755±0.036	4.403	0.038
α index	5.047±0.432	6.135±0.286	3.972	0.048
β index	10.235±0.861	12.447±0.570	4.131	0.044
PWVβ (m/s)	7.470±0.297	8.327±0.197	5.191	0.024

ANCOVA, the analysis of covariance; with age as covariate, age = 47.06.

Furthermore, NA-subjects were also categorized into four groups in terms of the number of risk factors: ‘zero’ (*n* = 47), ‘single’ (*n* = 21), ‘double’ (*n* = 47), and ‘multiple’ (*n* = 30), and the same ANVOCA was utilized. Table 4 lists ^RF^QIMT, 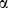, 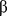, 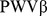, 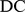, and 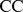 for all the four groups and their statistical correlation values. There was a significant difference in ^RF^QIMT among the four groups (*F* = 5.844, *p* = 0.01). ^RF^QIMT of the ‘multiple’ group was greater than in ‘zero’, ‘single’ or ‘double’ groups. The 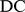 of the ‘zero’ group was significantly greater than in ‘single’, ‘double’ or ‘multiple’ groups (*F* = 2.924, *p* = 0.036). Note that 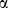 and 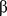 were significantly different among the groups (*p*<0.05). The 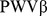 in ‘multiple’ group were significantly greater than in other three groups (*F* = 4.057, *p* = 0.008). However, there was no significant difference in 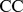 among the four groups (*F* = 1.975, *p* = 0.121).

**Table 4:** The ANCOVA of ^RF^QIMT, DC, CC, α, β, PWVβ in four groups of non-atherosclerosis subjects (^MAX^IMT<1.0 mm).

	Zero(*n* = 47)	Single(*n* = 21)	Double(*n* = 47)	Multiple(*n* = 30)	F value	*p* value
^RF^QIMT (mm)	0.554±0.018†	0.54±0.025 ˇ	0.611±0.017§	0.660±0.022‡	5.844	0.01
DC (1/kPa)	0.024±0.002	0.019±0.003	0.018±0.002†	0.015±0.002‡	2.924	0.036
CC (mm^2^/kPa)	0.905±0.055	0.835±0.075	0.723±0.051†	0.740±0.065	1.975	0.121
α index	4.952±0.428‡	4.952±0.583*	6.389±0.402†	6.714±0.508 ˇ	3.238	0.024
β index	10.05±0.854‡	10.10±1.162*	12.96±0.801†	13.59±1.013 ˇ	3.284	0.023
PWVβ (m/s)	7.399±0.294‡	7.489±0.4 ˇ	8.425±0.276†	8.872±0.348	4.057	0.008

ANCOVA, with age as covariate, age = 47.06.

† *p*<0.05 Zero vs Double;

‡ *p*<0.05 Zero vs Multiple;

§ *p*<0.05 Single vs Double;

*p<0.05 Single vs Double;

ˇ *p*<0.05 Single vs Multiple;

ξ *p*<0.05 Double vs Multiple.

Table 5 presents the results of the multiple linear regression analysis on independent risk factors for the ^RF^QIMT and carotid stiffness. Age (*p*<0.001), SBP (p = 0.003) and dyslipidemia (*p* = 0.019) were significant predictors of ^RF^QIMT. Age (*p*<0.001) and dyslipidemia (*p* = 0.004) were identified as significant predictors of 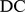. For 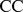, age (*p*<0.001) and SBP (*p* = 0.003) were identified as significant predictors. Age (*p*<0.001) and PP were significant predictors of 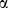 and 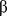. Age (*p*<0.001) and SBP (*p*<0.001) were significant predictors of 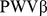. In summary, age is an important determinant of ^RF^QIMT and arterial elasticity.

**Table 5:** The stepwise multiple linear regression analysis of independent risk factors for the ^RF^QIMT and carotid stiffness.

Variables	Coefficient (β)	Standard Error	95% CI	*t* value	*p* value
^RF^QIMT (mm)					
Constant	–0.022	0.090	–0.199–0.155	–0.246	0.806
Age (years)	0.504	0.001	0.005–0.008	7.395	<0.001
SBP (mmHg)	0.200	0.001	0.001–0.004	3.024	0.003
Dyslipidemia	0.147	0.024	0.010–0.106	2.378	0.019
DC (1/kPa)					
Constant	0.037	0.003	0.031–0.043	12.140	<0.001
Age (years)	–0.361	0.000	–0.0004–0.0002	–4.900	<0.001
Dyslipidemia	–0.213	0.002	–0.009– −0.002	–2.888	0.004
CC (mm^2^/kPa)					
Constant	1.924	0.212	1.505–2.342	9.078	<0.001
Age (years)	–0.333	0.002	–0.013– −0.005	–4.266	<0.001
SBP (mmHg)	–0.235	0.002	–0.009– −0.002	–3.009	0.003
α index					
Constant	–1.398	1.054	–3.481–0.685	–1.326	0.187
Age (years)	0.398	0.018	0.058–0.129	5.190	<0.001
PP (mmHg)	0.202	0.022	0.015–0.104	2.632	0.009
β index					
Constant	–2.945	2.103	–7.099–1.028	–1.401	0.163
Age (years)	0.403	0.036	0.120–0.262	5.296	<0.001
PP (mmHg)	0.207	0.045	0.033–0.210	2.714	0.007
PWVβ (m/s)					
Constant	–1.883	1.175	–4.204–0.438	–1.602	0.111
Age (years)	0.421	0.011	0.047–0.091	6.222	<0.001
SBP (mmHg)	0.346	0.011	0.033–0.075	5.123	<0.001

## Discussion

Our statistical analysis results showed that carotid ^RF^QIMT and ^RF^QAS parameters can be used to estimate the degree of atherosclerosis in patients with or without risk factors. On one hand, measurements of carotid IMT in a clinical setting can identify individuals with advanced subclinical atherosclerosis and can quantify its severity noninvasively [20]. Our results in Table 2 showed good statistical correlations between ^MAX^IMT and ^RF^QIMT. For the NA-subjects Table 3 further shows that ^RF^QIMT in subjects with risk factors were significantly larger than in subjects without risk factors. On the other hand, the ultrasound RF-tracking technology provides several standard parameters for assessing arterial stiffness, including 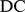, 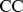, 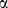, 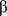 and 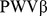. From Table 2 it can be seen that these parameters all have statistical correlations with ^MAX^IMT. From the comparisons between subjects with or without risk factors in Table 3, the observed significant increase for subjects with risk factors in values of 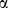, 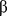 and 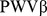 indicated an increase in arterial stiffness, and significant decrease in values of 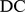 and 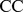 in the same subjects inferred a loss of arterial elasticity. This may have originated from smooth muscle cell proliferation, deposition of lipid, and accumulation of collagen, elastin, and proteoglycans without compensatory development of scar collagen at the early stage of atherosclerosis [21], [22]. We hypothesized that the multi-parameters obtained from the ultrasound RF-tracking technology can be used together with the routine IMT criteria to favor the diagnosis of subclinical atherosclerosis.

Also, abundant information that can be acquired from the ultrasound RF-tracking technology can assist in the differentiation of NA-subjects with different numbers of risk factors. The synergism of multiform risk factors is considered to accelerate the developmental process of atherosclerosis, and these risk factors were independently associated with increased carotid IMT and stiffness [23]. To the best of our knowledge, our studies for the first time employed the ultrasound RF-tracking technology to statistically evaluate the correlations between the number of risk factors and carotid IMT and stiffness for subjects without atherosclerotic symptoms. Table 4 shows that ^RF^QIMT got larger as the number of risk factors increased from zero, to single, double, and multiple. Also, 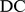, 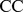, 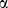, 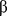 and 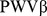 statistically varied with an increasing number of risk factors. Therefore, the ^RF^QIMT and ^RF^QAS parameters non-invasively acquired from the ultrasound RF-tracking technology do have significance for determining the grade of risk for subclinical atherosclerosis.

In addition, the stepwise multiple linear regression analysis on independent risk factors in Table 5 demonstrated age to be a significant predictor for all the stiffness parameters. This is consistent with independent prior studies that have proven that alterations in arterial structure and function occur with advancing age [24]. These changes comprised an increase in arterial wall thickness secondary to hyperplasia of the intima, addition of medial lamellae, and loss of the orderly arrangement of elastin in the media. Additionally, there was degeneration and disorganization of the medial layers with partial replacement of elastin with less compliant forms of collagen [24]. These changes in the arterial wall with advancing age are accompanied by a progressive dilation of major arteries and a progressive increase in arterial stiffness.

Moreover, from a technical point of view the ultrasound RF-tracking technology has its own advantages over *B*-mode-based ultrasound techniques. The RF signal is generally much richer in information compared to *B*-mode image. So, the ultrasound RF-tracking technology is not affected by non-linear post-processing steps such as log-compression and proprietary filter algorithms that modify the statistics of ultrasound signals for reasons of improved visual appeal. It also has desirable statistical properties facilitating various ways of distributional modeling of ultrasound specific texture patterns, referred to as speckle noise. It is worth noting that most reported studies measure IMT in individuals to the nearest 0.1 mm or even 0.01 mm, which may be beyond the spatial resolution limit of the ultrasound system [25]. For instance, the theoretical axial resolution of an ultrasound system with 4 to 13 MHz transducers is about 0.1 mm. The usage of pulses with multiple cycles in practice could degrade the axial resolution to be approximately 0.3 mm. The difference between “control” and “risk factor” groups in many observational studies of carotid IMT has been <0.2 mm [5], [26]. Other than counting on the sole criterion of IMT, the combination of IMT and arterial stiffness information can potentially improve the diagnosing capability of atherosclerosis, and the ultrasound RF-tracking technology holds promise for early detection of atherosclerosis. Although the ultrasound RF-tracking technology is currently not yet available for all commercial ultrasound devices, it will certainly be more accessible and gain importance in the clinical diagnosis of cardiovascular diseases in the near future.

The main limitation of this study is that we did not investigate intra-observer reliability and inter-observer reproducibility. But previous studies have indicated that the ultrasound RF-tracking technology can be used to assess the properties of the arterial wall with very high precision and reproducibility and real-time feedback on measurement quality [11], [14], [17]. A second limitation is the comparatively small number of subjects, especially older healthy volunteers. In this study, age was treated as a covariate to balance its possible influence on the statistical analysis. Further study with larger populations needs to be carried out to verify our results.

## Conclusions

We used the ultrasound RF-tracking technology to non-invasively acquire the human carotid IMT and carotid artery stiffness parameters from 160 subjects, of which 145 were non-atherosclerosis subjects with ^MAX^IMT smaller than 1.0 mm. We performed statistical analyses between these parameters and the number of risk factors for atherosclerosis, and found that ^RF^QIMT, 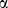, 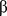 and 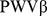 significantly increase and 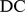 and 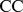 significantly decrease with an increasing number of risk factors. Further studies will be necessary to confirm the results of the present study and establish the diagnostic accuracy of this technique.
